# Coronoid impingement syndrome: literature review and clinical management

**DOI:** 10.1186/s40902-017-0111-7

**Published:** 2017-05-05

**Authors:** Priti Acharya, Andrew Stewart, Farhad B. Naini

**Affiliations:** 1 Eastman Dental Hospital, London, UK; 2 Kingston and St George’s Hospitals, London, UK

**Keywords:** Coronoid impingement syndrome, Coronoid process hyperplasia, Coronoidectomy

## Abstract

**Background:**

This case report discusses the unusual presentation of limited mouth opening as a result of bilateral coronoid process hyperplasia.

**Case presentation:**

A 14.5-year-old male patient of white Caucasian ethnicity presented with limited mouth opening, mandibular asymmetry, and dental crowding. Investigations confirmed bilateral coronoid process hyperplasia and management involved bilateral intraoral coronoidectomy surgery under general anaesthesia, followed by muscular rehabilitation. Mouth opening was restored to average maximum opening within 4 months of surgery.

**Conclusion:**

Limited mouth opening is a common presentation to medical and dental professionals. The rare but feasible diagnosis of coronoid impingement syndrome should not be overlooked.

## Background

Coronoid process hyperplasia is defined as ‘an abnormal elongation of the coronoid process, formed of histologically normal bone’ [1]. This unusual condition is relatively uncommon but well reported in the literature [1–10]. Coronoid hyperplasia was first reported by von Langenbeck in 1853 [11]. A review of case notes at the Queen Victoria Hospital, East Grinstead, over a 20-year period revealed 31 recorded cases, 23 of which were bilateral [2].

### Clinical presentation

The condition typically presents as painless progressive reduction in mouth opening due to contact interference between the elongated coronoid process and the medial surface of the zygomatic arch or the temporal aspect of zygomatic bone [2]. It can occur in unilateral and bilateral forms [9]. Facial asymmetry may occur if the hyperplasia is unilateral [11]. The condition is alternatively termed ‘coronoid impingement syndrome’ (CIS) [3]. Where pathological elongation of the coronoid process results in the formation of a new joint with the zygomatic process, this is also referred to as Jacob’s disease—named after Oscar Jacob in 1899 [11]. Symptoms can present as young as almost 7 years of age [2] and typically affect young patients with an average age of 25 years [7, 11]. A case of Jacob’s disease has been reported in a 39-year-old [10] and 52-year-old woman [11]. Male individuals are more commonly affected with a reported male to female ratio of 5:1 [12].

The aetiology of coronoid hyperplasia is as yet unclear [4, 10]. Possible causative factors include previous trauma to the temporomandibular joint, temporal muscle hyperactivity, chronic disc displacement, endocrine anomalies, and genetic alterations [7, 9, 11, 13]. Individuals with idiopathic short stature (ISS) who underwent treatment with growth hormone therapy have been found to develop trismus caused by bilateral coronoid process hyperplasia [7]. Familial inheritance patterns have been postulated [9]. Syndromic associations include trismus pseudocamptodactyly syndrome—affected individuals are unable to extend their fingers at the interphalangeal joints with their wrists in dorso-flexion and have severely limited mouth opening due to shortening of the temporalis muscle’s flexor muscle-tendon unit with possible coronoid process elongation. This syndrome is autosomal dominant, with variable expression [9].

Diagnosis can be made with panoramic radiographs [9, 10] and three-dimensional computerized tomography (CT) scans [5, 10, 14, 15]. Open mouth CT scans are useful for demonstrating direct impingement of the coronoid process on the zygoma [15]. Where limited mouth opening ability is observed, coronoid process elongation should always be considered as a possible aetiological factor [8].

### Histopathology

Histopathological examination is required to confirm a definitive diagnosis and reveals normal bone. The presence of cartilage and a synovial capsule indicates a new joint has formed, as in the case of Jacob’s disease [11].

### Differential diagnosis

Unilateral coronoid osteomas and osteochondromas are often mistaken for unilateral coronoid hyperplasia [16]. Histopathological examinations of surgical coronoidectomy specimens of cases with Jacob’s disease have revealed an osteochondroma of the affected coronoid process [10]. A rare case of bilateral coronoid process hyperplasia associated with nevoid basal cell carcinoma syndrome (also known as Gorlin-Goltz syndrome) has been reported [17].

### Management

Initial case management should always include a thorough history and clinical examination. Basic dental radiography including dental panoramic tomographs (DPTs) will reveal the outline of the mandible and relative size of the coronoid processes in relation to the condyles.

Where mouth opening is restricted to the extent that normal function is compromised, surgical treatment is often indicated [2, 3]. Surgery involves a coronoidectomy via an intraoral approach [6, 10], followed by early post-operative physiotherapy to prevent post-surgical fibrosis and re-establish muscular activity and maximum opening [1, 3, 6, 7, 10]. The use of dynamic laser physiotherapy post-surgery has also been suggested [10]. In the long term, patients should be monitored for possible regrowth of the coronoid process [7]. One case series report showed stable long-term results at 5 years post-surgery [6].

## Case presentation

A 14.5-year-old male patient of white Caucasian ethnicity (EP) presented to the Oral and Maxillofacial Surgery team, complaining of limited mouth opening and dental crowding. He reported functional and social difficulties associated with his limited mouth opening, and he was unable to have orthodontic treatment due to the same reason. His secondary concerns were an asymmetry of the right side of his lower jaw and constant dull headaches, which were interfering with his school attendance. He reported a noticeable reduction in his mouth opening from the age of 13 years, which coincided with his pubertal growth spurt. His mother and General Dental Practitioner also noticed this.

**Fig. 1:**
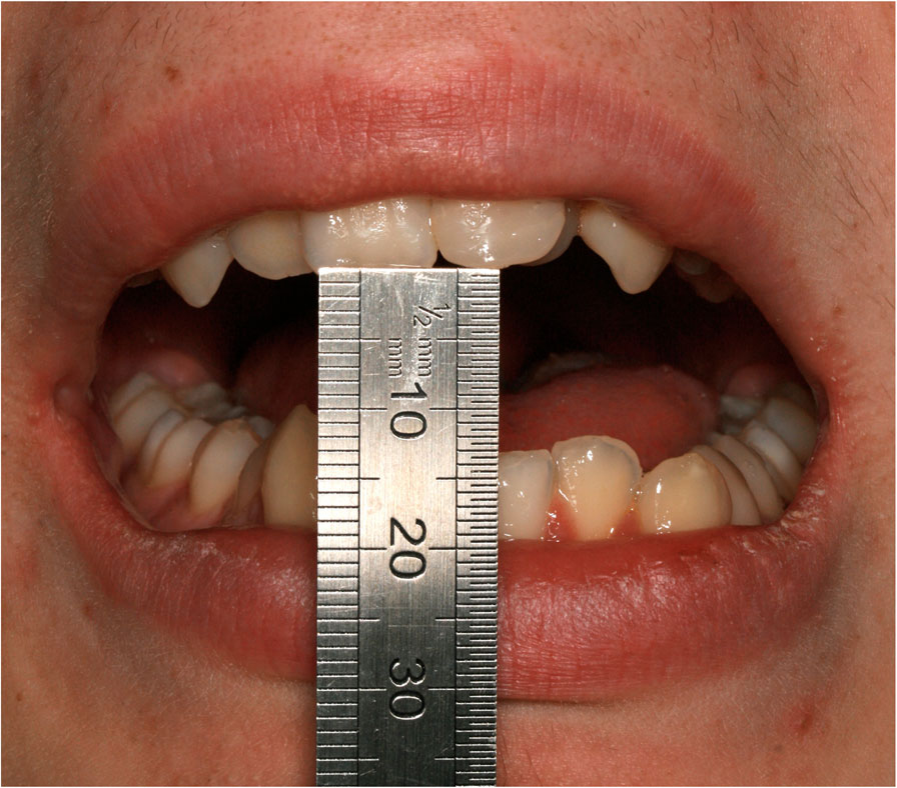
Preoperative frontal view demonstrating restricted mouth opening

**Fig. 2:**
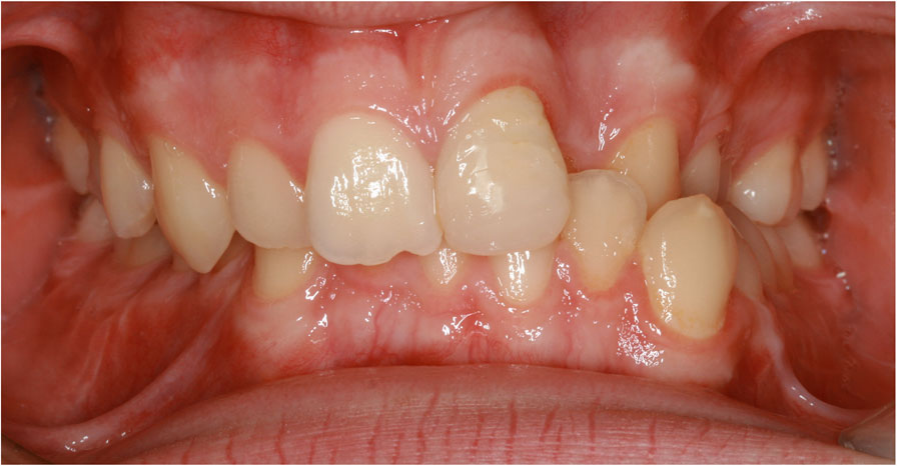
Intraoral frontal view

**Fig. 3:**
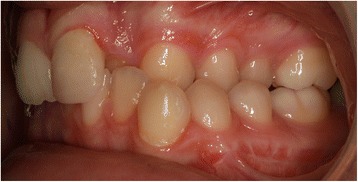
Intraoral left lateral view

**Fig. 4:**
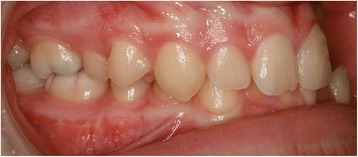
Intraoral right lateral view

**Fig. 5:**
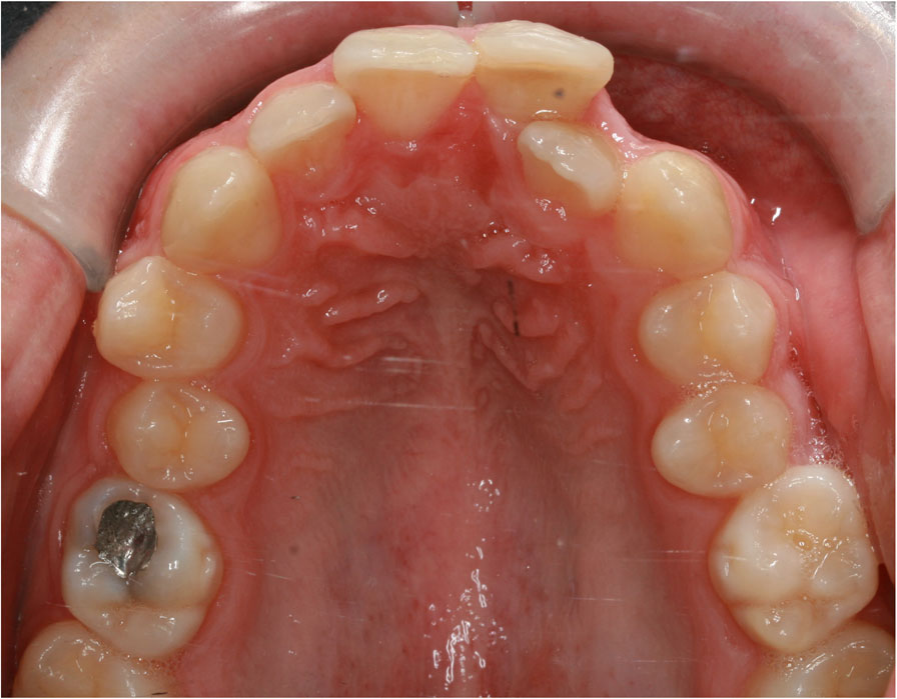
Intraoral maxillary occlusal view

**Fig. 6:**
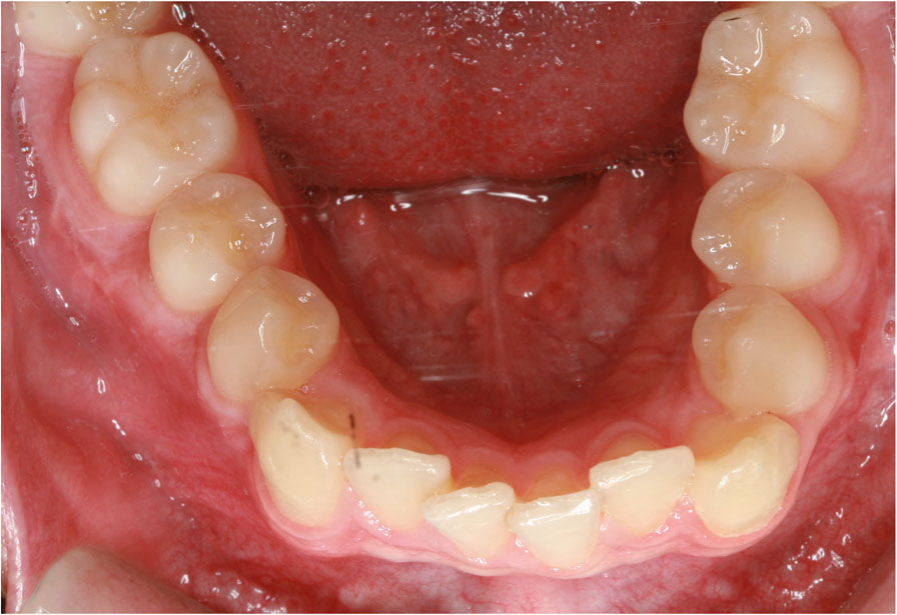
Intraoral mandibular occlusal view

EP presented with a Class II division 2 incisor relationship on a moderate Class II skeletal base with a chin point deviation to the left of his facial midline and an average lower anterior face height and Frankfort-mandibular plane angle. His maximum opening when assessed at age 15 years and 9 months was 15 mm between the maxillary and mandibular incisor teeth. Intraorally, he was in the adult dentition with all teeth erupted except his third molars. He had anterior dental crowding with dental centre line shifts and a deep impinging but atraumatic overbite. His right premolars were in scissor bite, and he had a scissor bite on the left side, associated with an anterior mandibular displacement which deviated to the left, in order to achieve maximum intercuspation. His oral hygiene was good, considering his limited mouth opening (Figs. 1, 2, 3, 4, 5, and 6).

**Fig. 7:**
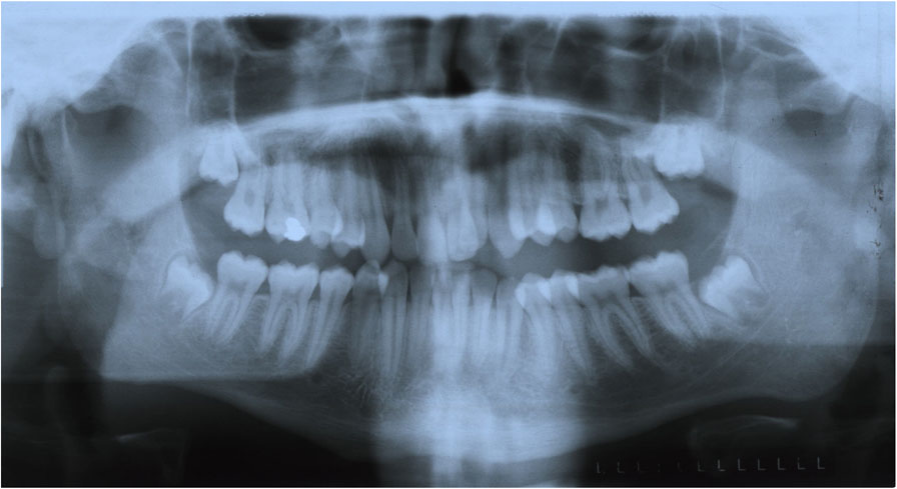
Presurgical orthopantomograph

**Fig. 8:**
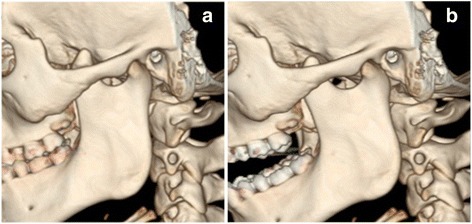
CT scan reconstructions of enlarged left coronoid process in **a** closed and **b** open jaw position

A dental panoramic tomograph revealed prominent bilateral mandibular coronoid processes (Fig. 7). Magnetic resonance imaging (MRI) scans revealed no obvious pathology of his temporomandibular joints. CT scans taken in a closed and open mouth position confirmed the presence of bilateral elongated coronoid processes with apparent impingement between the coronoid processes and zygomatic arches and the presence of bilateral pseudoarthrosis between the prominent coronoid process and the internal surface of the zygoma, as viewed in the parasagittal plane (Fig. 8a, b). Both temporomandibular joint complexes were morphologically normal with slightly underdeveloped condylar processes and a noted absence of expected movement of the condyles or discs in the open mouth position.

**Fig. 9:**
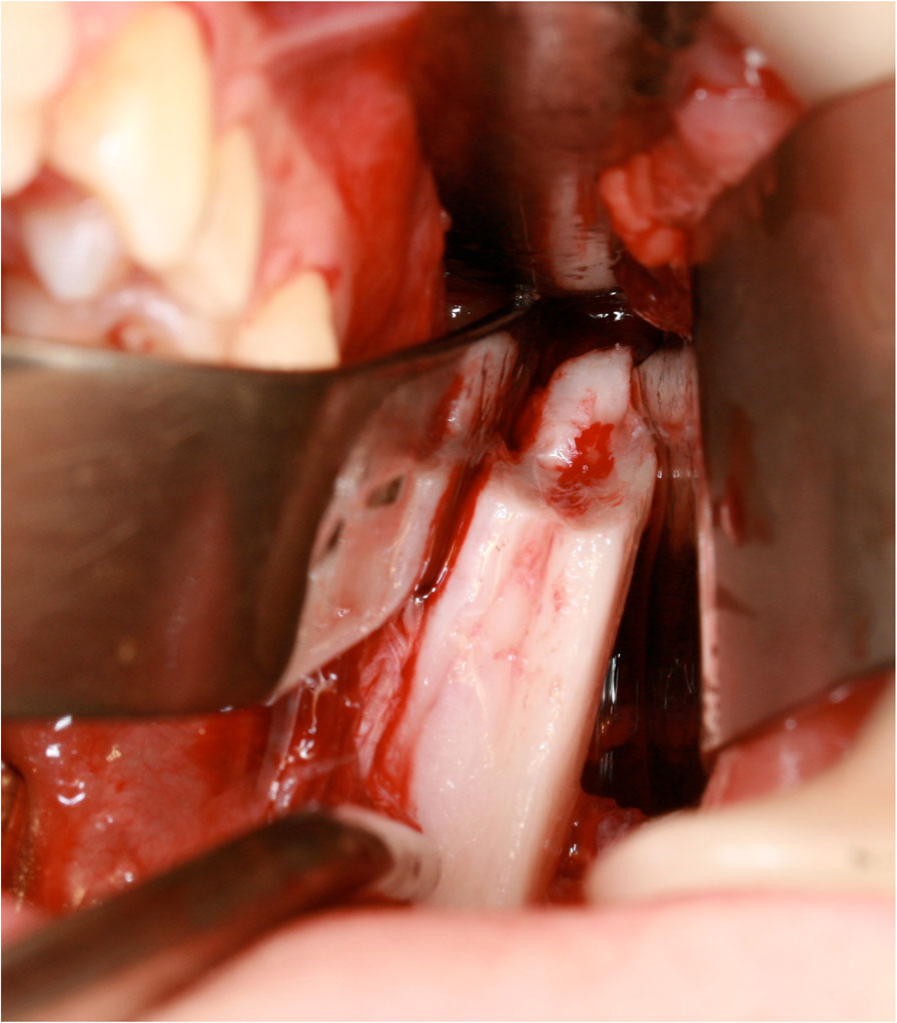
Coronoid process surgically removed

**Fig. 10:**
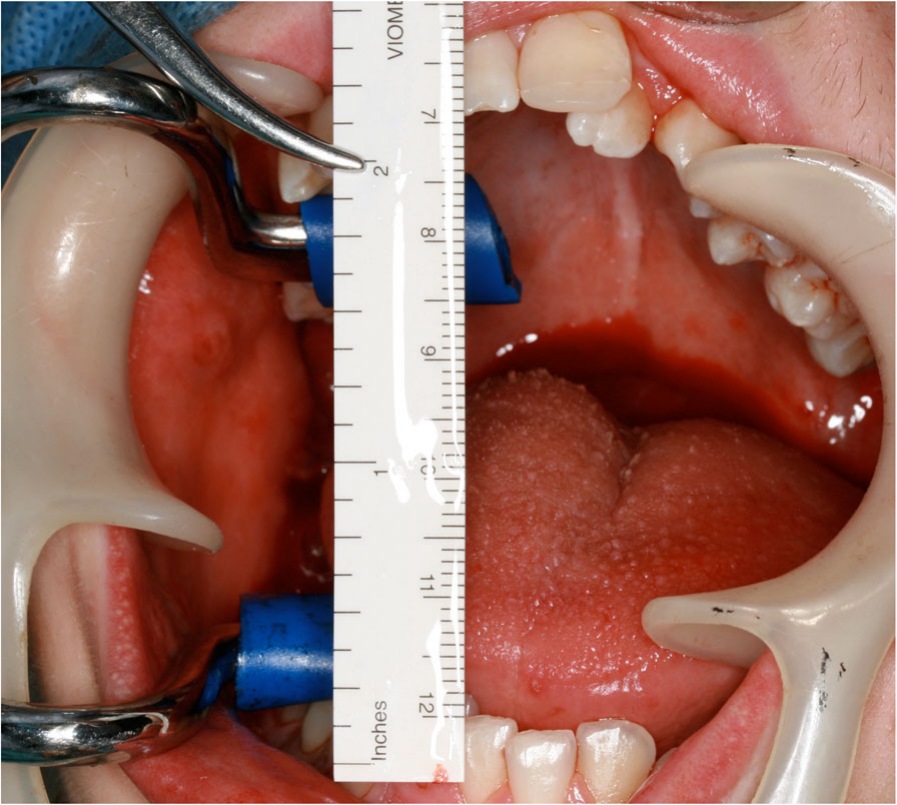
Improved mouth opening assessed immediately following surgery

**Fig. 11:**
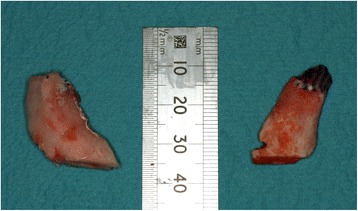
Surgically removed coronoid processes

**Fig. 12:**
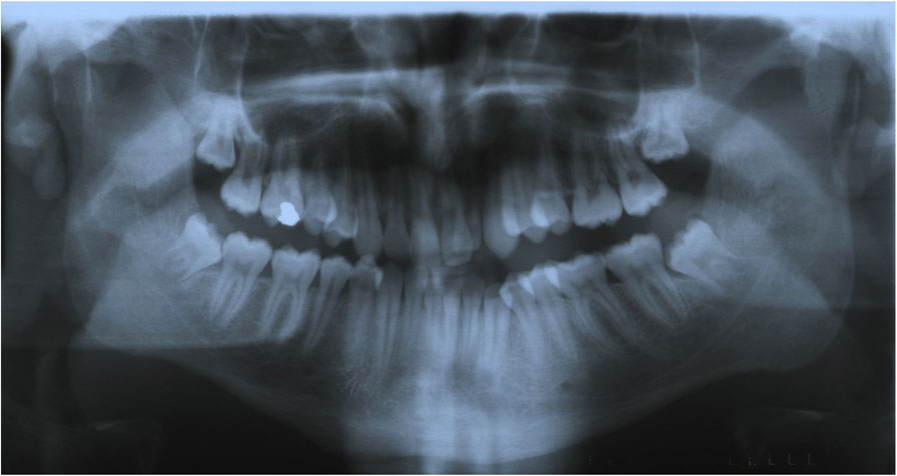
Post-surgical orthopantomograph demonstrating surgically removed coronoid processes

The patient was consented for bilateral coronoidectomy surgery via an intraoral approach to address his limited mouth opening. This was carried out when he was 15 years and 11 months old (Figs. 9, 10, 11, and 12).

Post-operative rehabilitation was largely facilitated by the use of the TheraBite® (registered trademark of Atos Medical AB, Sweden). This is an easy-to-use manual physiotherapy device which the patient places within their mouth passively and then activates to stretch their muscles of mastication to increase mandibular opening and mobility. The main indication is to improve mouth opening caused by soft tissue fibrosis (scar tissue) post-surgically [1].

**Fig. 13:**
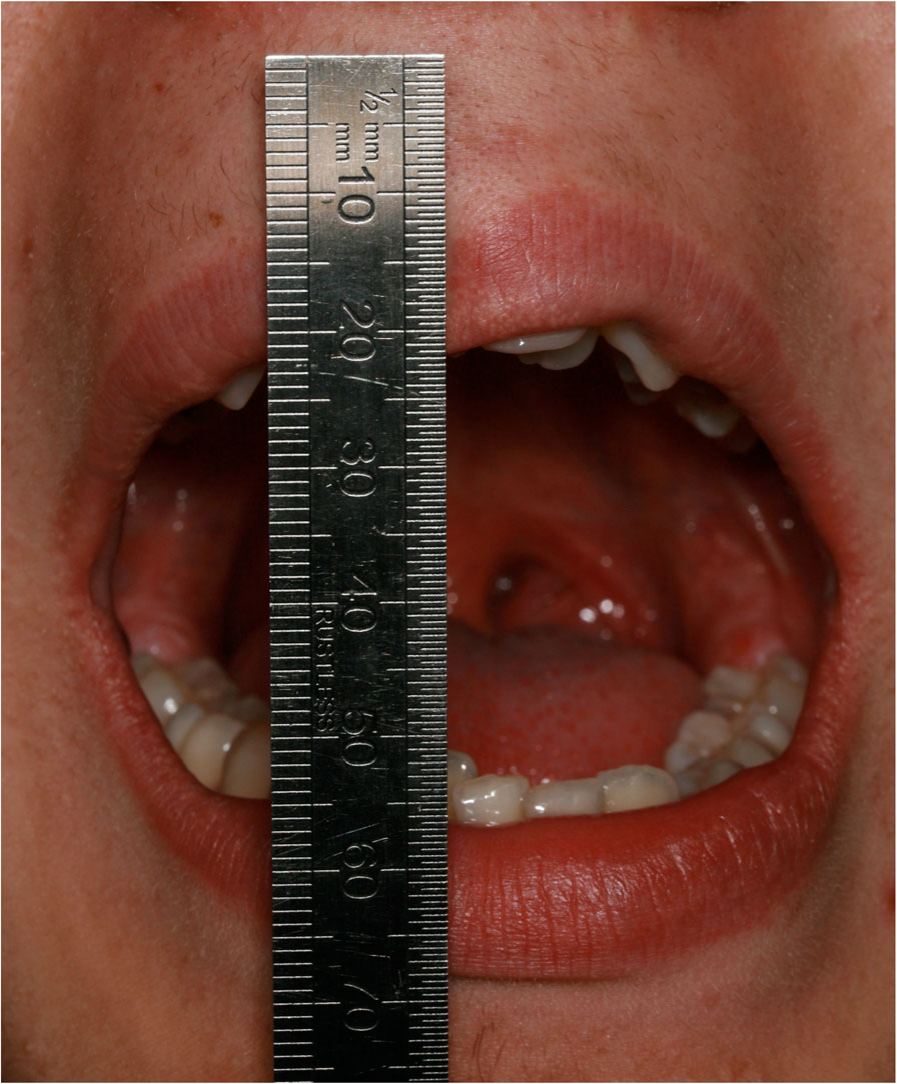
Normal mouth opening following healing phase

At 2 months, a significant increase in interincisal distance was noted, improved to 26 and 27 mm. The importance for continued jaw exercises was emphasized, and the use of the TheraBite device was checked at every review appointment. At his 3-month post-surgical review, EP reported that his occlusion felt more comfortable and he could comfortably open his mouth. His unassisted and assisted maximum mouth opening was 32 and 33 mm, respectively (Fig. 13). He had recently discontinued the use of the TheraBite device.

Following the completion of his post-surgical physiotherapy, EP expressed a wish to pursue orthodontic correction of his malocclusion. Orthodontic reassessment and planning was undertaken, and his orthodontic treatment carried out. His maximum mouth opening remains unchanged.

### Discussion

The TheraBite physiotherapy device has previously been successfully used in the post-surgical rehabilitation of a patient with bilateral coronoid process hyperplasia. It consists of two opposing padded, horseshoe-shaped surfaces which distribute forces evenly across all contacting teeth when activated. This should technically minimize the risk of dental trauma and joint overloading due to force application. A physiotherapy regime which commenced between 3 and 7 days post-surgically and consisted of 10-min exercises performed three times per day and repeated over 3–6 months has been advocated by previous authors [1].

The patient described in this case report commenced using their TheraBite appliance 1 week post-operatively and was asked to adhere to a similar regime as advocated above. The patient ceased using his appliance approximately 3 months post-surgery when he could comfortably achieve the maximum opening provided by the TheraBite appliance without the need for additional forces. He reported using the appliance for a total of 45 min per day rather than the 30 min minimum advocated.

EP’s maximum mouth opening was regularly reviewed for 6 months post-surgery to ensure this did not relapse.

## Conclusions

Coronoid impingement syndrome caused by coronoid process elongation should always be considered as a possible differential diagnosis in patients with severely limited mouth opening. Initial diagnosis is possible with simple panoramic radiography and supported by CT scans. Treatment often involves coronoidectomy surgery and should be supplemented with early post-operative physiotherapy to prevent scar tissue formation and re-establish normal muscle physiology. Proper post-operate rehabilitation is fundamental to maintaining the increased mouth opening seen immediately post-coronoidectomy surgery and achieving a successful clinical outcome.
